# Development and Validation of a Deep Learning-Based Model Using Computed Tomography Imaging for Predicting Disease Severity of Coronavirus Disease 2019

**DOI:** 10.3389/fbioe.2020.00898

**Published:** 2020-07-31

**Authors:** Lu-shan Xiao, Pu Li, Fenglong Sun, Yanpei Zhang, Chenghai Xu, Hongbo Zhu, Feng-Qin Cai, Yu-Lin He, Wen-Feng Zhang, Si-Cong Ma, Chenyi Hu, Mengchun Gong, Li Liu, Wenzhao Shi, Hong Zhu

**Affiliations:** ^1^Department of Medical Quality Management, Nanfang Hospital, Southern Medical University, Guangzhou, China; ^2^Department of Infectious Diseases, Nanfang Hospital, Southern Medical University, Guangzhou, China; ^3^State Drug Clinical Trial Agency, The First Affiliated Hospital, Nanchang University, Nanchang, China; ^4^Digital China Health Technologies Corporation Limited, Beijing, China; ^5^Department of Oncology, The First Affiliated Hospital, University of South China, Hengyang, China; ^6^Department of Radiology, The First Affiliated Hospital, Nanchang University, Nanchang, China; ^7^Department of Infectious Diseases, The First Affiliated Hospital, Nanchang University, Nanchang, China; ^8^Nanfang Hospital, Southern Medical University, Guangzhou, China

**Keywords:** COVID-19, computed tomography, deep learning, disease severity, multiple instance learning

## Abstract

**Objectives:**

Coronavirus disease 2019 (COVID-19) is sweeping the globe and has resulted in infections in millions of people. Patients with COVID-19 face a high fatality risk once symptoms worsen; therefore, early identification of severely ill patients can enable early intervention, prevent disease progression, and help reduce mortality. This study aims to develop an artificial intelligence-assisted tool using computed tomography (CT) imaging to predict disease severity and further estimate the risk of developing severe disease in patients suffering from COVID-19.

**Materials and Methods:**

Initial CT images of 408 confirmed COVID-19 patients were retrospectively collected between January 1, 2020 and March 18, 2020 from hospitals in Honghu and Nanchang. The data of 303 patients in the People’s Hospital of Honghu were assigned as the training data, and those of 105 patients in The First Affiliated Hospital of Nanchang University were assigned as the test dataset. A deep learning based-model using multiple instance learning and residual convolutional neural network (ResNet34) was developed and validated. The discrimination ability and prediction accuracy of the model were evaluated using the receiver operating characteristic curve and confusion matrix, respectively.

**Results:**

The deep learning-based model had an area under the curve (AUC) of 0.987 (95% confidence interval [CI]: 0.968–1.00) and an accuracy of 97.4% in the training set, whereas it had an AUC of 0.892 (0.828–0.955) and an accuracy of 81.9% in the test set. In the subgroup analysis of patients who had non-severe COVID-19 on admission, the model achieved AUCs of 0.955 (0.884–1.00) and 0.923 (0.864–0.983) and accuracies of 97.0 and 81.6% in the Honghu and Nanchang subgroups, respectively.

**Conclusion:**

Our deep learning-based model can accurately predict disease severity as well as disease progression in COVID-19 patients using CT imaging, offering promise for guiding clinical treatment.

## Introduction

The coronavirus disease (COVID-19) is rapidly spreading worldwide, leading to a global crisis. The first outbreak was reported in Wuhan, China, in December 2019 (Zhu et al., 2020), and the World Health Organization declared it a pandemic on March 11, 2020 (Sohrabi et al., 2020). Its pathogen is the severe acute respiratory syndrome coronavirus 2 (SARS-CoV-2). As of April 10, 2020, there have been 1,521,252 confirmed cases and 92,798 reported deaths (World Health Organization, 2020). The clinical presentation of COVID-19 varies in severity from asymptomatic infection to mild illness to severe or fatal illness. According to recent literature, the mortality rate for severe COVID-19 patients is approximately 20 times higher than that for non-severe COVID-19 patients (Chen N. et al., 2020; Gong et al., 2020; Yang et al., 2020). Thus far, no antiviral drugs with definite efficacy for COVID-19 have been found, and the treatment is mainly supportive. Once the disease progresses to severe type, the patients, particularly the elderly, face a high risk of death. Therefore, early identification and triage of patients with a high risk of future development of severe COVID-19, thereby implementing closer monitoring and timely intervention to prevent the occurrence of severe status, are effective strategies for saving lives, alleviating the burden on the healthcare system, and reducing mortality.

In the field of artificial intelligence and medical big data analysis, using machine learning-based classification algorithms to automatically diagnose and judge the severity of disease and aid clinical decisions has always been important. Notably, deep learning, a complex machine learning approach that has emerged in recent years, performs much better on language and image recognition than previous related technologies (Gupta et al., 2015; Schmidhuber, 2015). It has been used to detect various imaging features of chest computed tomography (CT) (Depeursinge et al., 2015; Anthimopoulos et al., 2016) and has recently been widely applied to facilitate the diagnosis of COVID-19 (Santosh, 2020). Recently, Li et al. (2020a) reported that artificial intelligence could be used to distinguish COVID-19 from community-acquired pneumonia on a chest CT. Song et al. (2020) noted that deep learning enables accurate diagnosis of COVID-19 with CT images. Many similar investigations combining artificial intelligence and accurate diagnosis of COVID-19 have also flourished (Chen J. et al., 2020; Fu et al., 2020; Zheng et al., 2020). Thus, artificial intelligence could provide immense help in the diagnosis of COVID-19. However, its role in predicting COVID-19 severity and disease progression has been far from established but of profound clinical significance in the COVID-19 epidemic situation.

Currently, lesion annotation is necessary for most deep learning-based methods of disease diagnosis, particularly for disease detection in CT volumes. Considering the rapidly growing epidemic, significant effort is needed in terms of annotation by radiologists. Unfortunately, the current shortage of radiologists due to the urgent COVID-19 setting implies that annotation dependent algorithms may be insufficient for the present medical need. Thus, performing COVID-19 detection in an “unsupervised” manner while simultaneously maintaining good predictive accuracies is of huge importance. In this study, we established and verified a multiple instance deep learning model using CT imaging to predict disease severity and the risk of future development of severe COVID-19. This model is expected to assist doctors in performing clinical diagnosis and treatment.

## Materials and Methods

### Patient Cohort

The Medical Ethics committee of Nanfang Hospital of Southern Medical University approved this retrospective analysis. Written informed consent from all study participants was obtained. This study included patients from the People’s Hospital of Honghu and The First Affiliated Hospital of Nanchang University, who met the following selection criteria: (1) confirmed case of COVID-19 with positive tests for 2019-nCoV nucleic acid and compliance with the guideline of Diagnosis and Treatment Protocol for Novel Coronavirus Pneumonia (Trial Version 7)(National Health Commission, 2020) developed by the Chinese National Health Commission and the State Administration of Traditional Chinese Medicine; (2) availability of initial lung CT imaging on admission. The exclusion criteria included the following: (1) incomplete clinical data; (2) coinfection with other respiratory viruses; (3) discharge within 24 h after admission. A total of 408 patients, consisting of 303 patients in the Honghu cohort and 105 in the Nanchang cohort, were included in the final analysis. Clinical electronic medical records, nursing records, and radiological reports for all included patients were reviewed and collected. Two researchers reviewed the electronic medical records manually and independently. They recorded the disease severity at admission, the daily assessment of the disease severity after admission, and the progression from non-severe type to severe type of COVID-19.

### Definition

The disease severity spectrum of COVID-19 covers states from mild to critical, according to the guidelines for novel coronavirus pneumonia (National Health Commission, 2020). In this study, all patients were diagnosed and classified based on the following definitions. Patients with mild clinical manifestation as well as no indication of pneumonia in imaging reports were considered to have a mild disease. Patients with fever, respiratory symptoms, and confirmed pneumonia on imaging were considered to have moderate disease. Patients who met any of the following criteria were considered to have a severe disease: oxygen saturation ≤ 93% at rest; respiratory distress (≥30 breaths/min); obvious lesion progression in chest imaging within 24–48 h > 50%; arterial partial pressure of oxygen (PaO2)/fraction of inspired oxygen (FiO2) ≤ 300 mmHg. Patients with organ failure and respiratory failure were considered to have a critical disease.

Patients who maintained mild or moderate symptoms were assigned to the non-severe group. Those with critical or severe disease conditions on admission and those who had moderate or mild disease on admission but developed severe COVID-19 after admission were assigned to the severe group.

The event of severe illness-free survival was defined as disease progression and the censored subject was defined as no occurrence of endpoint event by the time of discharge. Severe illness-free survival was defined as the time from the diagnosis of COVID-19 to the occurrence of disease progression.

### CT Data Cohort Construction Criteria

All CT images were downloaded via a picture archiving and communication system. Considering that each patient had received several CT examinations during hospitalization, there were multiple slices for each series. The patterns of rows/columns, pixel spacing, slice count, and thickness distributions for the data from The People’s Hospital of Honghu and The First Affiliated Hospital of Nanchang University are shown in Supplementary Figures S1, S2, respectively. We built CT series selection criteria for patients based on the following: (1) the CT series of the first examination after admission; (2) the pixel spacing and slice thickness being greater than 0.1 cm; (3) the number of slices being > 30; (4) a minimum lung and mediastinum window.

### Overall Preprocessing of Raw CT Data

The overall preprocessing procedure for raw CT data was as follows. The window width and level were narrowed to 1600 and 600, respectively; a pixel value with <5 percentile was set to the value of 5 percentile; that with <95 percentile was set to the 95 percentile. All DICOM CT series were resampled to the target pixel ([z: 5.0, y: 0.77, x: 0.77], mm), yielding median values in all CT series. In the resampled CT volume, the patch was cropped to as large as 3 × 224 × 224 (z × y × x); then, each CT value for the patch was scaled to the section between [0, 1], and each patch CT value was normalized to a mean of 0.5 and a variance of 1 (Supplementary Figure S3).

### Multiple Instance Learning of the Residual Neural Network

**FIGURE 1:**
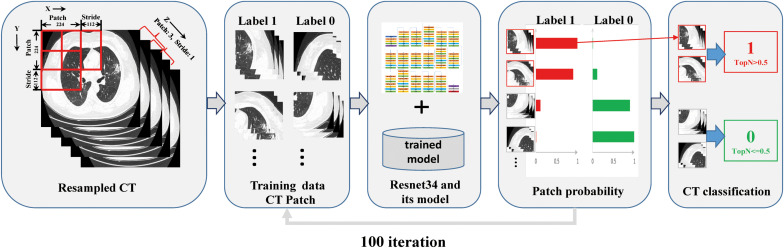
Overview of the multiple instance learning framework presented in this study. The patches cropped from resampled CT data were constructed for training, and patient-level prediction was performed via max-pooling of all patches belonging to the same one.

The residual neural network (ResNet34) is a representation of deep convolutional neural networks integrated with images, auto-encoding, and classification. In a prostate cancer pathology dataset using more than 10,000 slides, ResNet34 was found to perform better than other architectures as a backbone model for multiple instance learning (MIL) (Campanella et al., 2019). The architecture of ResNet34 is displayed in Supplementary Figure S4. The MIL framework was constructed based on the approach proposed by Campanella et al. (2019). In summary, the MIL training procedure was initiated by loading the patch dataset with a size as large as 3 × 224 × 224 (z × y × x) into the ResNet34 initialized with ImageNet pretrained weights, which were downloaded from https://download.pytorch.org/models/resnet34-333f7ec4.pth. The next steps included performing a full inference pass through the patch dataset, ranking the patches according to their probability of being positive or negative, and training the ResNet34 on the top four ranking patches from every three adjacent resampled CT slides. These three steps were repeated for 100 iterations, and a patient-level CT classification was generated via max-pooling of all the patches belonging to the same one (Figure 1 and Supplementary Figure S3).

### Training, Validation, and Testing

Five-fold cross-validation was performed with 17,336 CT images from the resampled CT data of 303 patients who had been treated at the People’s Hospital of Honghu, and 6,476 CT images from the resampled CT data of 105 patients at The First Affiliated Hospital of Nanchang University were used as the test dataset.

### Multiple Instance Learning Comparison Within Different Backbone Networks

There are several varieties of the network architecture for the ResNet series, including ResNet34 (Xie et al., 2017), AlexNet (Krizhevsky et al., 2012), VGGNet (Simonyan and Zisserman, 2014), and DenseNet (Huang et al., 2017). We conducted a set of comparison experiments with these networks using the CT images from Honghu and Nanchang. At least ten training runs were completed for each deep neural network. The performance metrics for different backbone networks are presented in Supplementary Table S1. We found that ResNet34 achieved the largest area under the curve (AUC) in both the Honghu and Nanchang cohorts compared with other networks, indicating the superiority of ResNet34 over other network architectures. For data imbalance, a class-weighted loss was performed to obtain better performance, and we adopted weights of 0.8 for severely ill patients and 0.2 for non-severe patients on admission.

### Classification Metrics

Seven general classification metrics, including sensitivity, specificity, positive prediction value (PPV), negative prediction value (NPV), false positive rate (FPV), false negative rate (FNR), accuracy, and AUC, were used to estimate the classifiers’ performance of all deep neural network models used for the training. Their methods of calculation are given in Equations (1) – (7).

(1) Sensitivity=TP/⁢(TP+FN)

(2) Specificity=TN/⁢(TN+FP)

(3) PPV=TP/⁢(TP+FP)

(4) NPV=TN/⁢(TN+FN)

(5) FPR=FP/⁢(TP+FN)

(6) FNR=FN/⁢(TN+FP)

(7) Accuracy=(TP+TN)/(TP+TN+FP+FN).

Here, TP is the true positive, TN is the true negative, FP is the false positive, and FN is the false negative; the AUC value was obtained using R statistical software.

### Implementation Details

Our implementation was based on the PyTorch (version 1.0) pretrained model for the ResNet34 network. Our training experiments were conducted in a Linux environment on a machine for data loading, building models, and training (CPU: Intel Xeon Processor Silver 4114 at 2.20 GHz; GPU: NVIDIA P Tesla V100; RAM: 128 GB).

### Code Availability

The source code and our best weights of the trained ResNet34 for this work can be downloaded from: https://github.com/dchealth/covid-mil.git.

### Statistical Analysis

Continuous variable data are presented as medians (interquartile ranges, IQR). Classified variable data are presented as n (%). Statistical analyses were performed using R statistical software version 3.5.0 (R Core Team, 2020). The receiver operating characteristic (ROC) curves were plotted with the pROC package (Robin et al., 2011). A confidence interval (CI) of 95% for AUC was calculated with the pROC package (Robin et al., 2011) using bootstrapping with non-parametric unstratified resampling, as described by Carpenter and Bithell (2000). The confusion matrices were plotted using Python 3.6 in two different cohorts to calculate the accuracy of the model prediction.

## Results

### Clinical Characteristics of Participant Patients

A total of 408 patients with confirmed COVID-19 were included in this study, with 303 patients in the Honghu cohort and 105 in the Nanchang cohort as the training set and test set, respectively. The demographics and baseline characteristics of the Honghu and Nanchang cohorts are listed in Table 1. The median ages were 49.0 (IQR 36.0–60.0), 45.0 (IQR 35.5–54.5), and 48.0 (IQR 36.0–58.0), and the numbers of males were 162 (53.5%), 43 (41.0%), and 205 (50.2%) in the Honghu cohort, Nanchang cohort, and all patients, respectively. Additionally, 48 (15.8%), 40 (38.1%), and 88 (21.6%) patients were assigned to the severe group in this study, respectively, among whom 12 (4.0%), 11 (10.5%), and 23 (5.6%) exhibited mild symptoms on admission but deteriorated to severe disease later.

**TABLE 1:** Demographics and baseline characteristics of Honghu and Nanchang cohorts.

**Characteristic**	**Honghu cohort (*n* = 303)**	**Nanchang cohort (*n* = 105)**	**All patients (*n* = 408)**
Age, years	49.0(36.0−60.0)	45.0(35.5−54.5)	48.0(36.0−58.0)
Male	162 (53.5)	43 (41.0)	205 (50.2)
**Smoking history**			
Current smokers	4 (1.3)	7 (6.7)	11 (2.7)
Ex-smokers	1 (0.3)	1 (1.0)	2 (0.5)
**Comorbidity**			
Any	61 (20.1)	26 (24.8)	87 (21.3)
Hypertension	42 (13.9)	13 (12.0)	55 (13.5)
Diabetes mellitus	14 (4.6)	11 (10.5)	25 (6.1)
Cardiovascular disease	8 (2.6)	0 (0.0)	8 (2.0)
Cerebrovascular disease	3 (1.0)	0 (0.0)	3 (0.7)
Chronic liver disease	2 (0.7)	9 (8.6)	11 (2.7)
COPD	2 (0.7)	0 (0.0)	2 (0.5)
Asthma	1 (0.3)	0 (0.0)	1 (0.2)
Renal disease	1 (0.3)	2 (1.9)	3 (0.7)
Cancer	2 (0.7)	0 (0.0)	2 (0.5)
**Signs and symptoms**			
Fever	175 (57.8)	82 (78.1)	257 (63.0)
Temperature on admission (°C)	36.7(36.5−37.0)	37.0(36.5−37.7)	36.8(36.5−37.1)
Highest temperature (°C)	37.0(36.5−38.0)	38.0(37.5−38.5)	37.3(36.6−38.0)
Cough	170 (56.1)	59 (56.2)	229 (56.1)
Sputum production	41 (13.5)	29 (27.6)	70 (17.2)
Nasal congestion	1 (0.3)	6 (5.7)	7 (1.7)
Fatigue	68 (22.4)	29 (27.6)	97 (23.8)
Headache	10 (3.3)	9 (8.6)	19 (4.7)
Sore throat	18 (5.9)	22 (21.0)	40 (9.8)
Shortness of breath	31 (10.2)	10 (19.5)	41(10.0)
Dyspnea	18 (5.9)	5 (4.8)	23 (5.6)
Anorexia	11 (3.6)	26 (24.8)	37 (9.1)
Diarrhea	13 (4.3)	5 (4.8)	18 (4.4)
Nausea	12 (4.0)	4 (3.8)	16 (3.9)
Myalgia or arthralgia	1 (0.3)	2 (1.9)	3 (0.7)
Combination of bacterial infection	206 (68.0)	64 (61.0)	270 (66.2)
**Laboratory findings**			
White blood cell count, × 10^9^/L	5.8(4.7−7.2)	5.0(3.6−6.7)	5.6(4.3−7.0)
Lymphocyte count × 10^9^/L	1.5(1.1−1.8)	1.0(0.7−1.4)	1.4(1.0−1.8)
Neutrophil count, ×10^9^/L	3.5(2.6−5.0)	3.5(2.1−5.1)	3.5(2.5−5.0)
Albumin, g/L	40.5(36.5−43.8)	43.6(40.0−46.4)	41.3(37.2−44.6)
Total bilirubin, μmol/L	10.7(7.8−13.7)	9.0(5.4−12.3)	10.0(7.4−13.5)
Direct bilirubin, μmol/L	2.9(2.3−4.1)	2.6(2.0−3.9)	2.9(2.2−4.1)
Alanine aminotransferase, U/L	22.0(15.0−37.0)	17.0(12.0−34.0)	21.0(14.0−36.0)
Gamma-glutamyl transferase, U/L	26.0(18.0−44.0)	23.0(14.0−45.0)	24.5(17.0−43.8)
Prothrombin time, s	12.7(12.0−13.2)	12.3(11.9−12.9)	12.6(12.0−13.2)
Creatinine, μmol/L	62.8(51.3−74.9)	64.8(51.4−79.3)	63.7(51.4−75.5)
Urea nitrogen, mmol/L	4.1(3.3−5.2)	4.1(3.4−5.3)	4.1(3.3−5.3)
Lactate dehydrogenase, U/L	205.5(169.0−252.0)	228.0(190.5−309.5)	209.0(175.0−260.0)
Creatinine kinase, U/L	65.0(43.0−96.0)	87.0(59.5−125.0)	68.0(46.0−110.0)
C-reactive protein, mg/L	2.3(0.5−16.1)	12.3(3.1−35.4)	3.3(0.5−21.9)
**Abnormalities on chest CT**			
Ground-glass opacity	164 (54.1)	48 (45.7)	212 (52.0)
Local patchy shadowing	47 (15.5)	13 (12.4)	60 (14.7)
Bilateral patchy shadowing	167 (55.1)	78 (74.3)	245 (60.0)
Interstitial abnormalities	14 (4.6)	0 (0.0)	14 (3.4)
Multi-lobular infiltration	201 (66.3)	89 (84.8)	290 (71.1)
**Treatment**			
Antiviral therapy	291 (96.0)	105 (100.0)	396 (97.1)
Antibiotic therapy	195 (64.4)	105 (100.0)	300 (73.5)
Use of corticosteroid	118 (38.9)	64 (61.0)	182 (44.6)
Oxygen support	98 (32.3)	105 (100.0)	203 (49.8)
**Clinical outcomes**			
Discharge from hospital	298 (98.3)	105 (100.0)	403 (98.8)
Length of hospital stay	15.0(10.0−22.0)	16.00(12.0−21.0)	15.5(10.0−21.0)
Death	5 (1.7)	0 (0.00)	5 (1.2)
Severe type	48 (15.8)	40 (38.1)	88 (21.6)
Mild to severe type	12 (4.0)	11 (10.5)	23 (5.6)

*Continuous variable data are presented as median (interquartile ranges, IQR). Classified variable data are presented as n (%).*

### Training and Validation of the Multiple Instance Learning Model in Disease Severity Prediction

**FIGURE 2:**
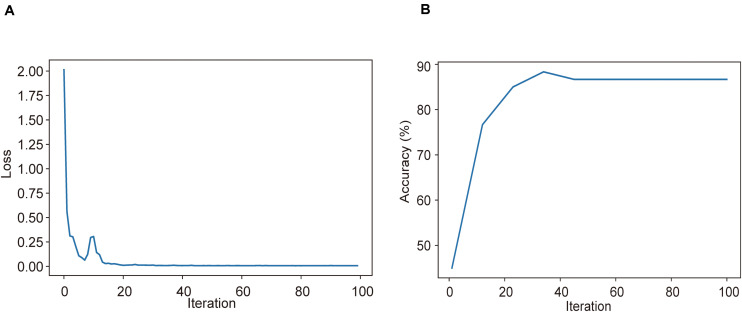
Training and validation processes of the deep learning model based on CT images for the task of disease severity prediction. **(A)** Cross-entropy loss and **(B)** accuracy were plotted against 100 training iterations. The cross-entropy loss was close to 0.03, and the final validation accuracy was 87%.

**FIGURE 3:**
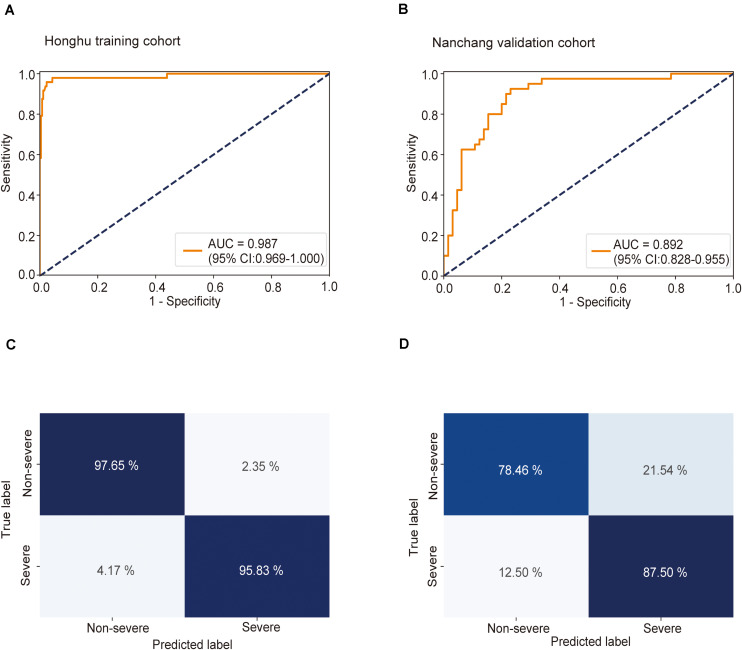
Receiver operating characteristic (ROC) curve and confusion matrix for predicting disease severity in the training and test sets. The prediction result of severity is shown via the ROC curve. **(A)** In the training set, the multiple instance learning model had an area under the curve (AUC) of 0.987 (CI: 0.967–1.00); **(B)** in the test set, the model had an AUC of 0.892 (CI: 0.828–0.955). Confusion matrix indicating the prediction quality of the multiple instance learning model classification for the **(C)** training and **(D)** test datasets.

Using CT imaging and ResNet34, a disease severity prediction model based on MIL was developed in the training cohort. The cross-entropy loss was close to 0.03 (Figure 2A) and the final accuracy was 87% for the 100 training iterations (Figure 2B). In the training set, the MIL model achieved an AUC of 0.987 (0.969–1.000) (Figure 3A). This model was also subjected to validation, revealing that the MIL model maintained its outstanding performance with an AUC of 0.892 (0.828–0.955) in the test set (Figure 3B). These results indicated the excellent discrimination capability of this model for disease severity prediction.

To “uncover” the black box and make our model more intuitive, we displayed slices associated with severe and non-severe COVID-19 cases from six randomly selected patients (three severe and three non-severe). It can be observed that disease severity is positively correlated with the area and density of lesions in lungs. For the severe cases (Supplementary Figures S5A–C), multiple large consolidations and ground glass density shadows were seen in both lungs. An air bronchial sign could be seen in the consolidation shadow. The lesions were extensive, mostly under the peripheral pleura, and a small amount of effusion could be seen in both sides of the chest. For the non-severe cases (Supplementary Figures S5D–F), small flakes and wedge-shaped ground glass density shadows could be observed under the pleura of the left lower lobe, with a clear boundary. Thickened small blood vessels could be seen in the left lung, with a normal shape. The maximum positive probability of all patches extracted from the CT series is assigned to the positive probability for the represented slices. If the positive probability for the represented slices is larger than 0.5, the corresponding case is predicted as severe; if it is smaller than 0.5, the case is predicted as non-severe. The predicted probability was in accordance with disease severity, highlighting the accuracy of our model in predicting disease severity.

Next, the predictive accuracy of the model was evaluated via a confusion matrix. In the training dataset, the model correctly discriminated against 97.7% of patients in the non-severe group and 95.8% of patients in the severe group (Figure 3C), with an accuracy of 97.4% and an error rate of 2.6%. In the test set, 78.5% of patients in the non-severe group and 87.5% of patients in the severe group were correctly identified by this model (Figure 3D). The accuracy and error rates were 81.9 and 18.1%, respectively. Other performance metrics were used, and the results are summarized in Supplementary Table S1. Besides, decision curve analysis (DCA) showed that our model achieved an outstanding overall net benefit in the training cohort and validation cohort (Supplementary Figure S6), indicating the clinical utility of our model in predicting COVID-19 severity.

### Clinical Significance of the Multiple Instance Learning Model in Predicting the Future Risk of Development of Severe COVID-19

The clinical significance of the predictive model lies in its ability to identify patients in the early stages of the disease who were mildly ill on admission but progressed to severe disease later. To further estimate the practical significance of the MIL model, we performed a clinically important subgroup analysis. Patients with severe symptoms on admission were excluded from both the Honghu and the Nanchang cohorts, whereas those who presented non-severe symptoms on admission were retained.

**FIGURE 4:**
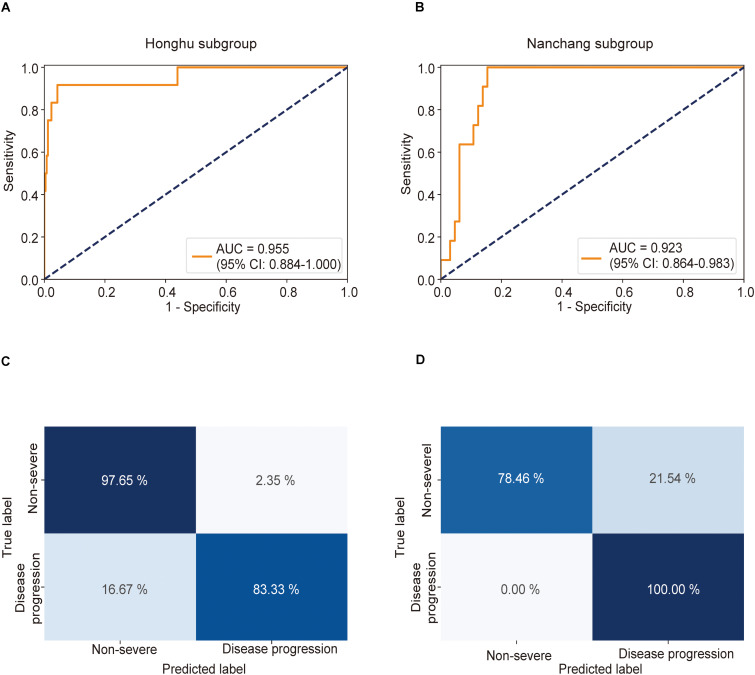
Receiver operating characteristic (ROC) curve and confusion matrix for predicting disease progression in the Honghu and Nanchang subgroups. The prediction result of disease progression is shown via the ROC curve. Patients who presented non-severe symptoms on admission in the Honghu and Nanchang cohorts were assigned to the Honghu and Nanchang subgroups, respectively. **(A)** In the Honghu subgroup, the multiple instance learning model had an area under the curve (AUC) of 0.955 (CI: 0.884–1.00); **(B)** in the Nanchang subgroup, the model had an AUC of 0.923 (CI: 0.864–0.983). Confusion matrix indicating the prediction quality of the multiple instance learning model classification for the **(C)** training and **(D)** test datasets.

This model achieved AUCs as high as 0.955 (0.884–1.000) (Figure 4A) and 0.923 (0.864–0.983) (Figure 4B) in the Honghu and Nanchang subgroups, respectively. Further, this model correctly predicted 97.6 and 78.5% of patients in the non-severe group and 83.3 and 100.0% patients in the severe group in the Honghu (Figure 4C) and Nanchang subgroups (Figure 4D), respectively. The accuracies were 97.0 and 81.6%, and the error rates were 3.0 and 18.4%, respectively. These results highlight the accuracy of our model in predicting disease progression.

To further demonstrate the clinical significance of our model, we performed DCA and clinical prognosis analysis. DCA revealed that our model achieved an excellent overall net benefit in the Honghu and Nanchang subgroups (Supplementary Figure S7), which is of significant clinical implication for predicting the risk of future development of severe COVID-19. The result suggests that our model would be a good choice for clinical use. Moreover, Kaplan–Meier curves were generated based on the prediction results and severe illness-free survival was taken into account. Patients were stratified into a high-risk group when the probability of disease progression was higher than 0.5 and assigned to a low-risk group when the probability was less than or equal to 0.5. Compared with the low-risk group, the high-risk group had a significantly lower survival probability in both the Honghu and Nanchang subgroups (Honghu: *P* < 0.001; Nanchang: *P* < 0.001, log-rank test) (Supplementary Figure S8). These results indicate that our model can be employed as a useful tool for prognosis prediction, representing an important advancement in AI-assisted clinical management. In general, the results presented above confirmed the potential practicability of our model in clinical practice.

## Discussion

In this retrospective study, we developed and validated a MIL-based predictive model using CT imaging of 408 patients to enable accurate identification of patients with severe COVID-19. Subgroup analyses in patients with non-severe COVID-19 on admission revealed that this model could accurately predict disease deterioration in the early stages. To our knowledge, this is the first study to demonstrate a novel application of MIL to establish a disease severity predictive model for COVID-19. This model has the potential to contribute to computer-aided diagnosis and guide clinical treatment by preemptively identifying patients who have a high risk of experiencing more severe symptoms.

Many models have been developed to facilitate early identification of patients with a high risk of progression. The parameters used in these models are mainly clinical indicators and imaging findings, which are known for their role in the diagnosis and prognosis of COVID-19 as specified by the Chinese guideline for COVID-19 (National Health Commission, 2020). Predictive models mainly based on clinical indicators are easy to use and popular among physicians. For instance (Liang et al., 2020), screened epidemiological, clinical, laboratory, and imaging variables obtained from hospital admission and constructed a predictive risk score (COVID-GRAM), which provided physicians with an important tool for improving the clinical care of patients with the worst disease outcomes, including admission to ICU and death. Compared with clinical indicators, CT imaging has important advantages of intuitiveness and non-invasiveness while being fast and yielding relatively stable results. A CT scanning procedure needs a shorter turnaround time than a molecular diagnostic test. The latter is performed in a standard laboratory and has a relatively long workflow. Additionally, CT imaging can provide radiologists with more detailed information associated with the pathology and natural course of COVID-19 (Wang et al., 2020; Zheng, 2020) and is favorable for quantitative assessment of the lesion size, lesion type, and the severity or extent of lung involvement, with crucial prognostic implications. From the clinical application perspective, predictive tools based on CT imaging as well as on chest X-rays to enable accurate diagnosis of COVID-19 have flourished. Mukherjee et al. (2020) established a convolutional neural network combined with chest X-rays for COVID-19 outbreak screening. Rajinikanth et al. (2020) proposed a Harmony Search and Otsu-based system to detect COVID-19 using CT scan images. However, the use of CT imaging for predicting disease progression is not as developed as its use for disease diagnosis. Therefore, based on the advantages of previous theories and technologies, extending the diagnostic role of CT imaging to predicting the future development of severe COVID-19 holds a great promise.

At present, there are two main methods for predicting the risk of progression using chest CT. In one method, radiologists manually assign a score for each image to identify patients with potential severe disease, represented by the “CT severity score,” whereas the other method utilizes artificial intelligence to automatically calculate the risk of progression.

The CT severity score in the first method (Chang et al., 2005) is a semiquantitative index for estimating the involvement of the lung, which is related to the number of involved lobes and the extent of the lesions: the larger the score, the higher the risk of future development of severe COVID-19. The effectiveness of the CT severity score in the early prediction of COVID-19 progression was validated by some COVID-19 studies (Feng et al., 2020; Li et al., 2020b; Xu et al., 2020; Zheng, 2020). In addition, the CT severity score has also been proposed for prognosis and mortality prediction in acute Middle East respiratory syndrome (Das et al., 2015). However, the final score varies under different radiologists, as it is dependent on expertise and experience, resulting in the volatility of the prediction. More importantly, this time-consuming task would aggravate the overall workload of radiologists in an epidemic situation when an increasing number of patients are waiting for risk screening for severe COVID-19, constraining the wide application of the CT severity score.

Conventional supervised learning in the artificial intelligence-assisted method requires labeling information and lesion annotation for each object to guide learning of the algorithm. On the one hand, labeling is slow and laborious, and radiologists working in a COVID-19 setting do not have sufficient time to label every slice of a CT image. On the other hand, it is difficult for radiologists to mark the lesion area very accurately; thus, both the normal and pathological tissues could inevitably exist in the marked area, resulting in a large amount of noise in the training set that consequently affects the accuracy of learning. However, the MIL method adopted in this study differs from a supervised machine learning technique. It requires only patient-level labels to inform the algorithm whether the patient would progress to a severe symptom. Labels on each chest CT image and annotation of the lesion area are not needed in this method, thereby saving radiologists precious time and improving the accuracy of the algorithm to some extent.

In this section, the overall advantage of our model in clinical practice is highlighted. Our model exploited the superiority of CT imaging, making the prediction procedure fast, intuitive, and non-invasive. Compared with the CT severity score, our model enabled a relatively objective and accurate diagnosis of severe COVID-19, which increased reliability and improved quality control. Our model can maintain the same diagnostic criteria anywhere and ensure that the AUCs are as high as 0.987 and 0.892, with accuracies of 97.4 and 81.9% in the training and test sets, respectively. Compared with other supervised AI-assisted predictive models, our AI system eliminates the huge workload of annotating the lesion, allowing radiologists to save time and increasing their capacity to handle emergencies quickly and effectively, while maintaining high accuracy. Easing the workload of radiologists in terms of lesion annotations as well as quick and accurate prediction of the procedure will facilitate risk predictions in daily practice, which is extremely important for COVID-19 management owing to the severe limitations that occur with regard to healthcare resources.

There are several limitations to this study. First, our sample size was relatively small, which would inevitably result in some bias. Additional patient data is required to validate our model. Moreover, a disadvantage of all deep learning methods is the lack of transparency and interpretability. More efforts to determine the correlation between clinical processes and the prediction results of our model should be made in the future.

## Conclusion

We employed MIL, a deep learning method, using quantitative CT data to accurately predict the disease severity of COVID-19. By utilizing an inexpensive and widely available test, our model can be used to identify patients at high risk of disease progression in the early phase of the disease, which has important practical implications for conducting early intervention, preventing disease progression, and reducing mortality. We recommend that confirmed COVID-19 patients should undergo CT screening as soon as they are admitted to the hospital, so that physicians can use our model to determine the risk of severe disease. If the result indicates a potential worsening of the condition of the patient, closer monitoring and early intervention should be considered before the disease severity increases. We hope that this model would be of some help to clinicians to better manage patients and contribute to combatting COVID-19.

## Data Availability Statement

Reasonable data requests are welcome. A proposal and detailed illustration of the study aims will be needed to assess the reasonability of requests. After approval from participating hospitals and the corresponding authors, de-identified clinical data will be provided.

## Ethics Statement

The studies involving human participants were reviewed and approved by the Medical Ethics committee of Nanfang Hospital of Southern Medical University. The patients/participants provided their written informed consent to participate in this study.

## Author Contributions

HZ had full access to all the data in the study and took responsibility for the integrity of the data and accuracy of the data analysis. LX, PL, FS, YZ, and CX contributed equally to this work. LX, LL, and WS contributed to concept and design. F-QC, Y-LH, W-FZ, MG, LL, and LX acquired, analyzed, and interpreted the data. LX, FS, YZ, and CX drafted the manuscript. PL, HBZ, and S-CM critically revised the manuscript for important intellectual content. LX, FS, CX, and CH contributed to the statistical analysis. HZ and LL obtained the funding. WS, MG, F-QC, Y-LH, and W-FZ contributed to administrative, technical, or material support. HZ, WS, and LL supervised the study. All the authors contributed to the article and approved the submitted version.

## Conflict of Interest

FS, CX, MG, and WS were employed by the Digital China Health Technologies Corporation Limited (Beijing, China). The remaining authors declare that the research was conducted in the absence of any commercial or financial relationships that could be construed as a potential conflict of interest.

## Supplementary Material

FIGURE S1 Distributions of features for raw CT data from the People’s Hospital of Honghu. Row/columns, pixel spacing, slice count, and thickness distributions for CT data from the People’s Hospital of Honghu.

FIGURE S2 Distributions of features for raw CT data from the First Affiliated Hospital of Nanchang University. Row/columns, pixel spacing, slice count, and thickness distributions for CT data from the First Affiliated Hospital of Nanchang University.

FIGURE S3 CT data preprocessing, training, and inference workflow.

FIGURE S4 Architecture of ResNet34 used for multiple instance learning.

FIGURE S5 Performance of our model in predicting disease severity in six cases. Slices from six randomly selected patients in the validation cohort [three severe **(A–C)** and three non-severe **(D–F)**] are shown. The maximum positive probability of all patches extracted from the CT series is assigned to the positive probability for the represented slices. If the positive probability for the represented slices is larger than 0.5, the corresponding case is predicted as severe; if it is smaller than 0.5, the case is predicted as non-severe.

FIGURE S6 Decision curve for the multiple instance learning (MIL) model for predicting disease severity in patients with confirmed COVID-19. Dotted line: the MIL model. Gray line: assumed all patients have severe COVID-19. Solid black line: assumed no patient has severe COVID-19. The decision curve provided the expected net benefit of three scenarios in predicting disease severity in the **(A)** Honghu training cohort and **(B)** Nanchang validation cohort.

FIGURE S7 Decision curve for the multiple instance learning (MIL) model for predicting disease progression in COVID-19 patients with non-severe symptoms on admission. Dotted line: the MIL model. Gray line: assumed all patients have severe COVID-19. Solid black line: assumed no patient has severe COVID-19. The decision curve provided the expected net benefit of three scenarios in predicting disease severity in the **(A)** Honghu subgroup and **(B)** Nanchang subgroup.

FIGURE S8 Severe illness-free survival curves for the high and low severe risk groups. Patients with the non-severe disease on admission were included in this analysis. Patients were stratified into a high-risk group when the probability of disease progression was higher than or equal to 0.5 and assigned to a low-risk group when the probability was less than 0.5. Kaplan–Meier curves exhibited a distinct difference in the survival probability in the **(A)** Honghu subgroup and **(B)** Nanchang subgroup. The *P*-value was calculated using the log-rank test.
