# Supplementary figures and images for: Development and Validation of a Deep Learning-Based Model Using Computed Tomography Imaging for Predicting Disease Severity of Coronavirus Disease 2019
Source: Front Bioeng Biotechnol. 2020 Jul 31;8:898. doi: 10.3389/fbioe.2020.00898 (PMC7411489; doi:10.3389/fbioe.2020.00898)

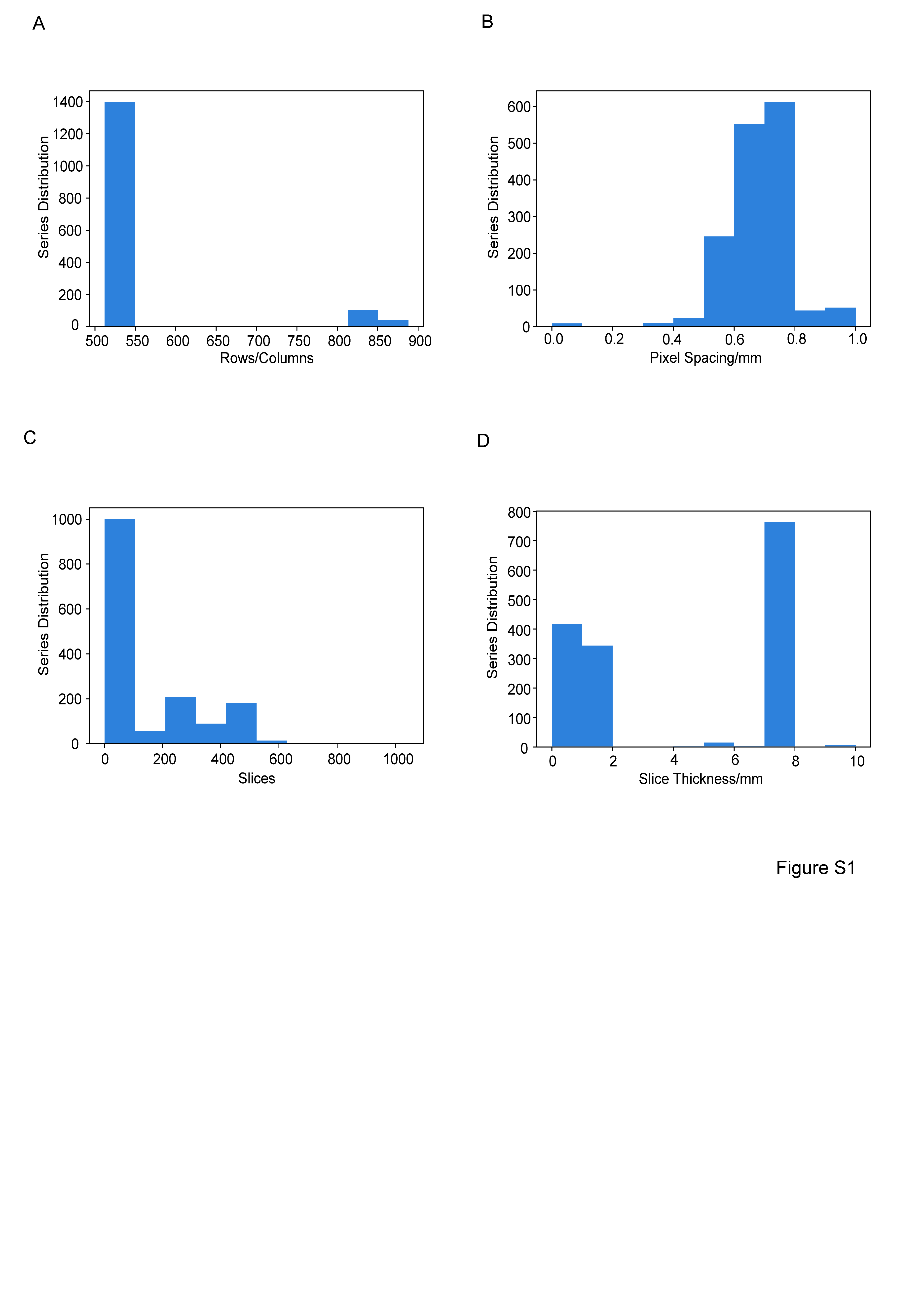

Supplement: FIGURE S1 — Distributions of features for raw CT data from the People’s Hospital of Honghu. Row/columns, pixel spacing, slice count, and thickness distributions for CT data from the People’s Hospital of Honghu. [file Image_1.tif]

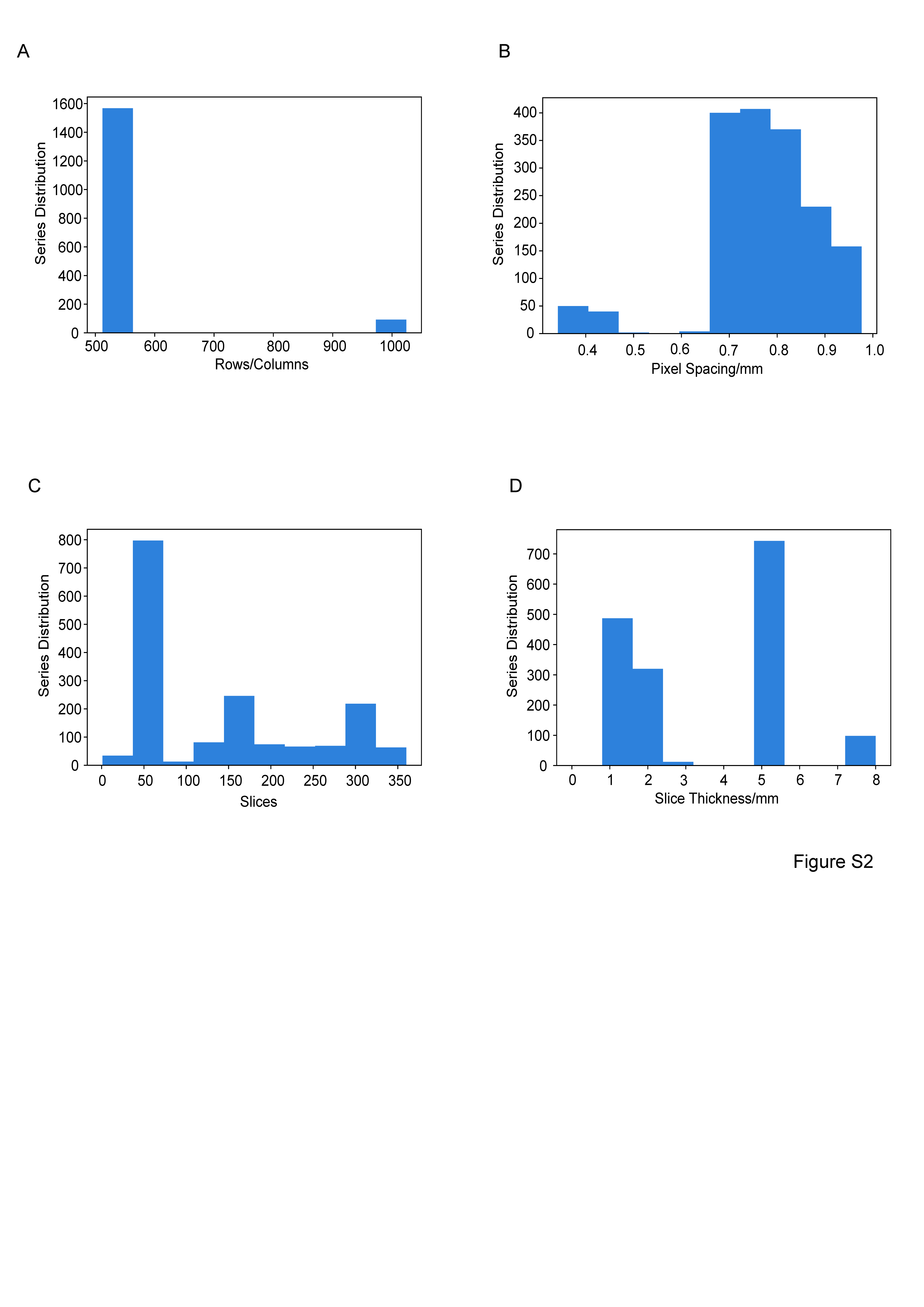

Supplement: FIGURE S2 — Distributions of features for raw CT data from the First Affiliated Hospital of Nanchang University. Row/columns, pixel spacing, slice count, and thickness distributions for CT data from the First Affiliated Hospital of Nanchang University. [file Image_2.tif]

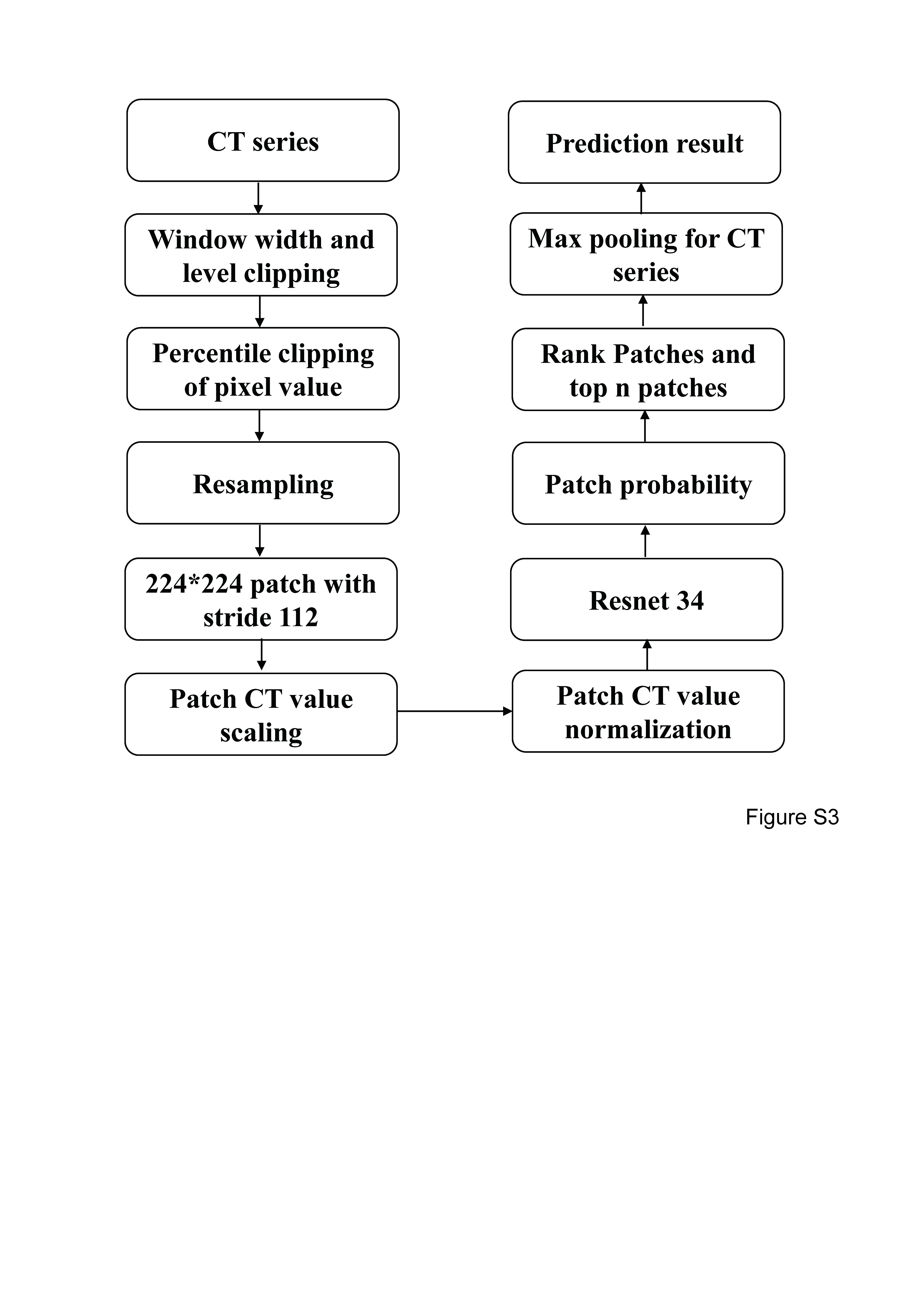

Supplement: FIGURE S3 — CT data preprocessing, training, and inference workflow. [file Image_3.tif]

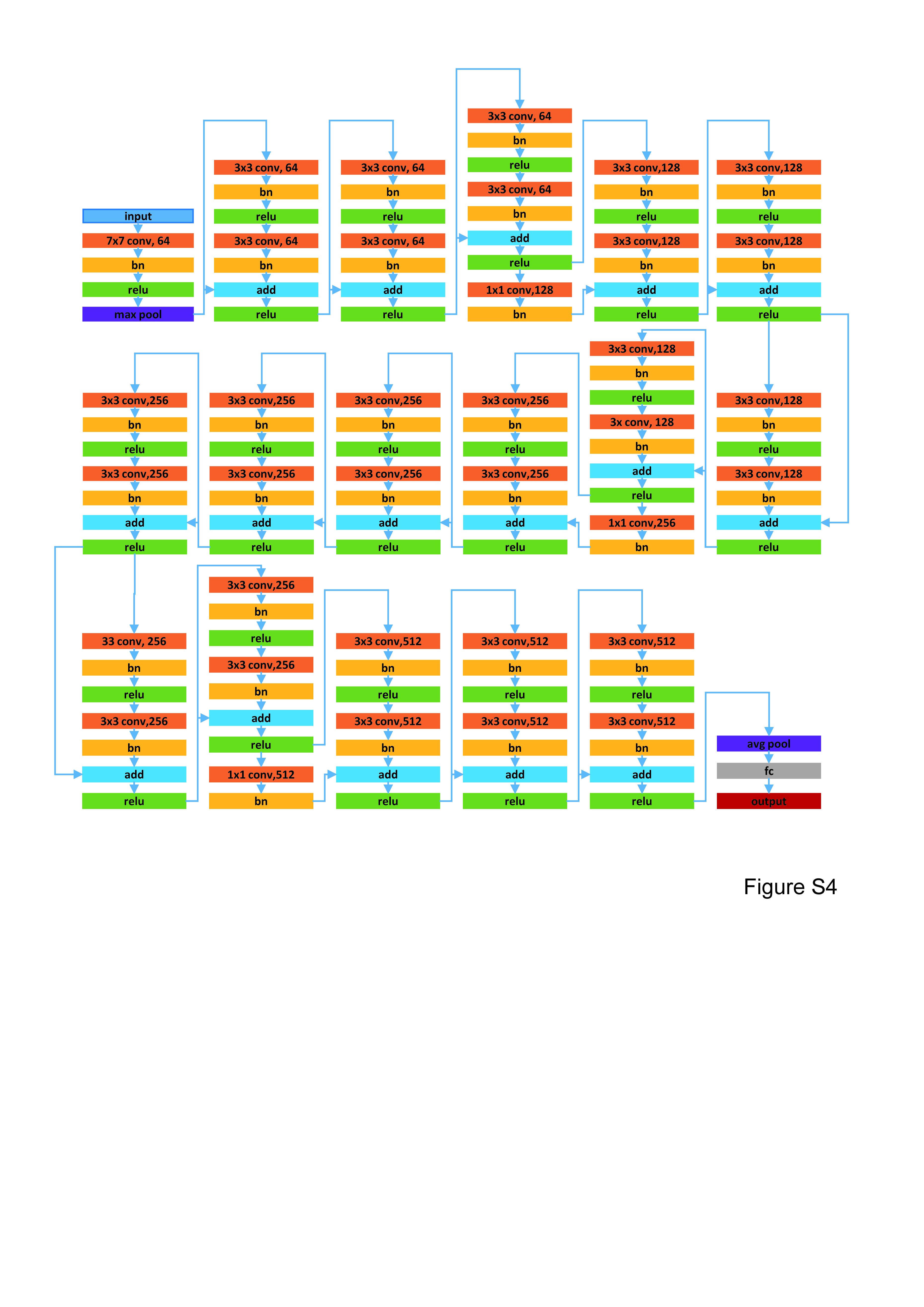

Supplement: FIGURE S4 — Architecture of ResNet34 used for multiple instance learning. [file Image_4.tif]

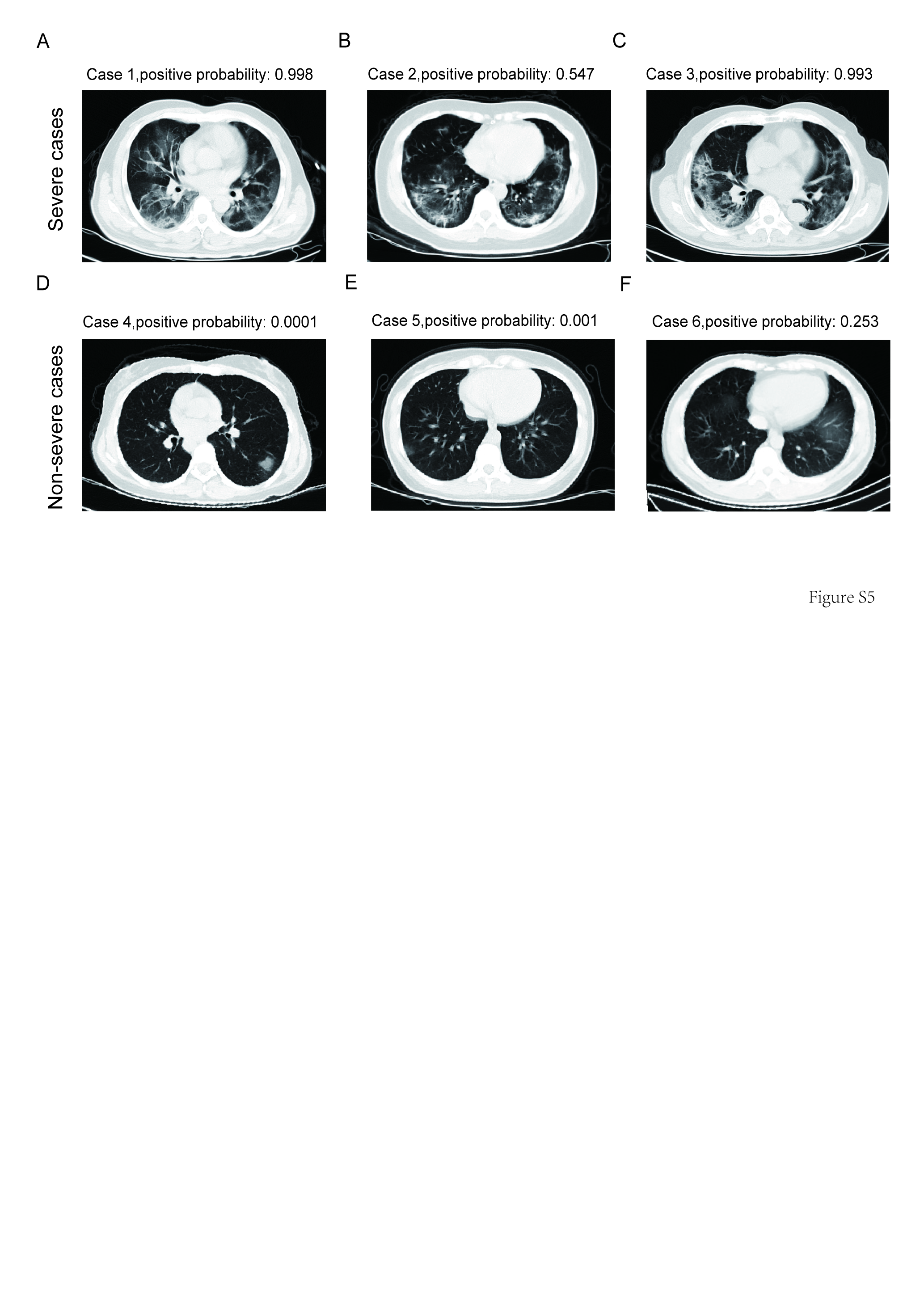

Supplement: FIGURE S5 — Performance of our model in predicting disease severity in six cases. Slices from six randomly selected patients in the validation cohort [three severe (A–C) and three non-severe (D–F)] are shown. The maximum positive probability of all patches extracted from the CT series is assigned to the positive probability for the represented slices. If the positive probability for the represented slices is larger than 0.5, the corresponding case is predicted as severe; if it is smaller than 0.5, the case is predicted as non-severe. [file Image_5.tif]

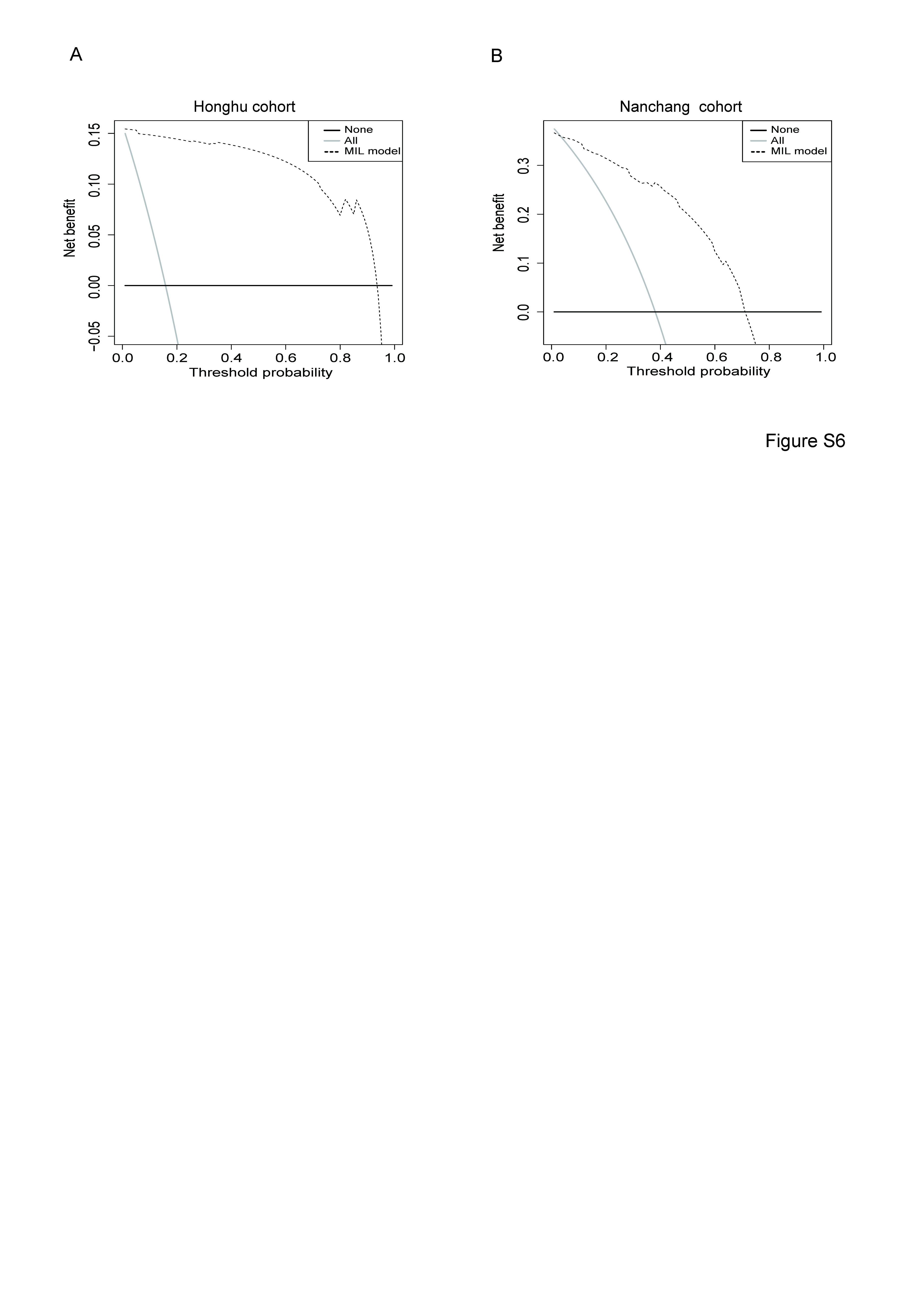

Supplement: FIGURE S6 — Decision curve for the multiple instance learning (MIL) model for predicting disease severity in patients with confirmed COVID-19. Dotted line: the MIL model. Gray line: assumed all patients have severe COVID-19. Solid black line: assumed no patient has severe COVID-19. The decision curve provided the expected net benefit of three scenarios in predicting disease severity in the (A) Honghu training cohort and (B) Nanchang validation cohort. [file Image_6.tif]

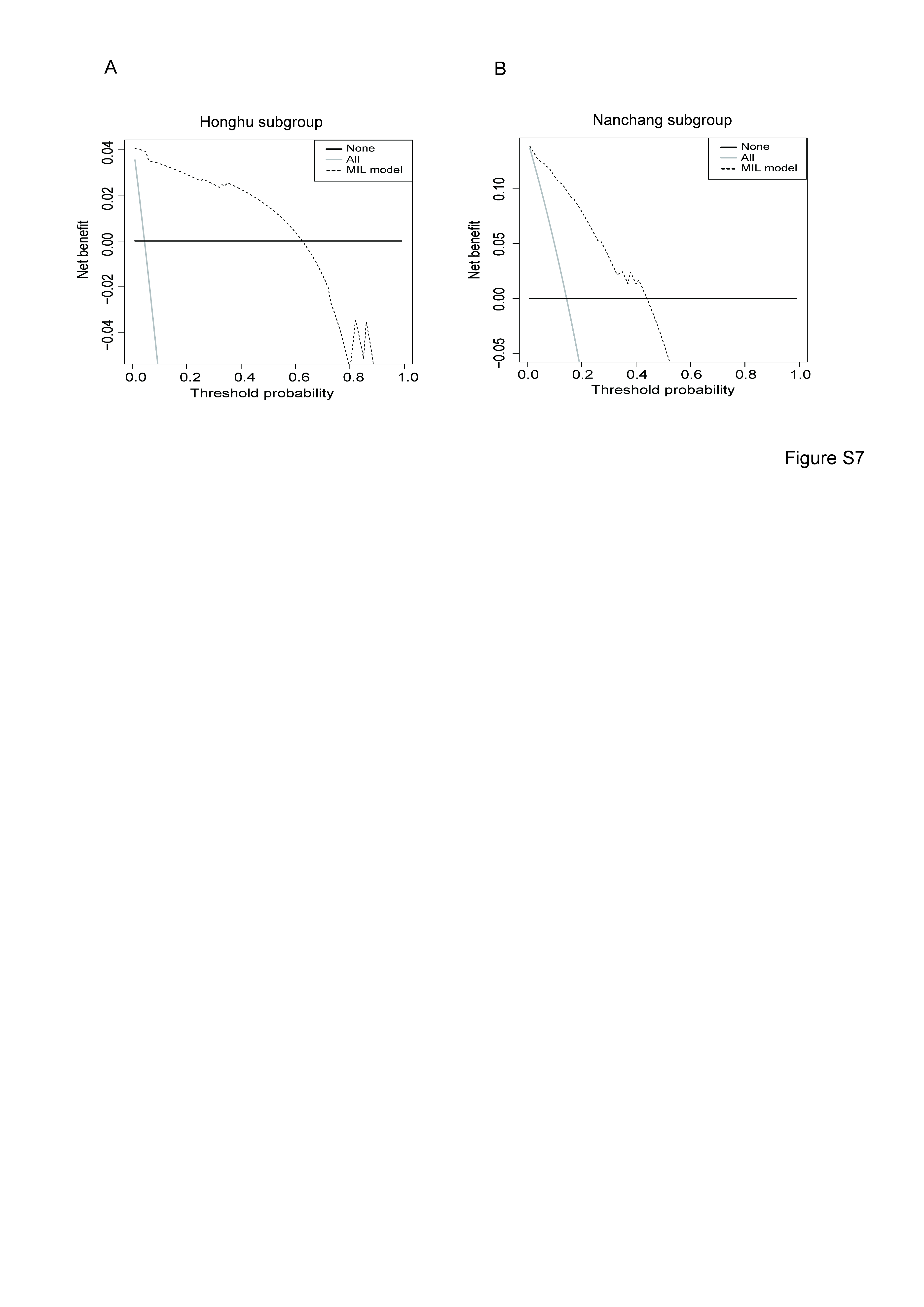

Supplement: FIGURE S7 — Decision curve for the multiple instance learning (MIL) model for predicting disease progression in COVID-19 patients with non-severe symptoms on admission. Dotted line: the MIL model. Gray line: assumed all patients have severe COVID-19. Solid black line: assumed no patient has severe COVID-19. The decision curve provided the expected net benefit of three scenarios in predicting disease severity in the (A) Honghu subgroup and (B) Nanchang subgroup. [file Image_7.tif]

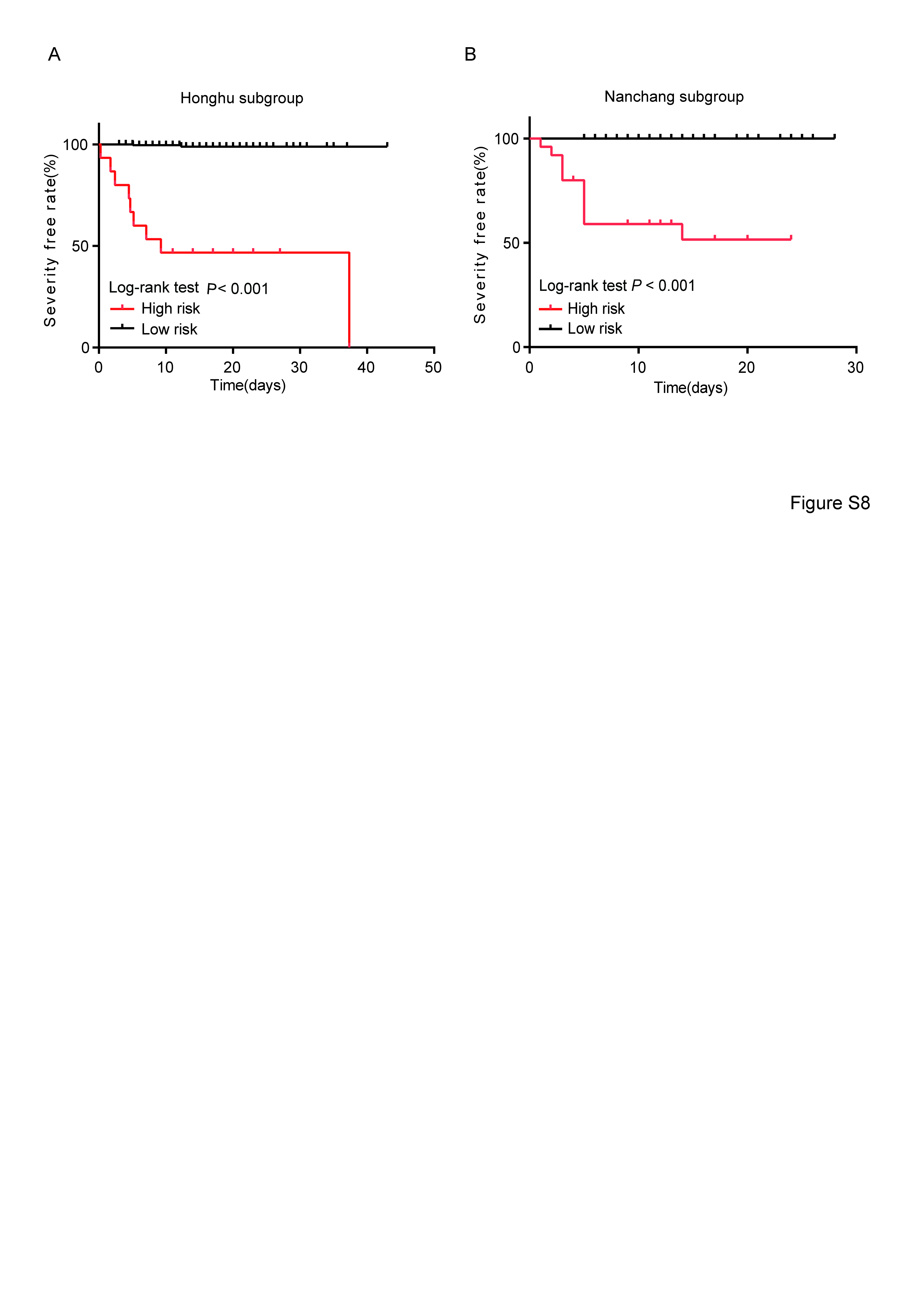

Supplement: FIGURE S8 — Severe illness-free survival curves for the high and low severe risk groups. Patients with the non-severe disease on admission were included in this analysis. Patients were stratified into a high-risk group when the probability of disease progression was higher than or equal to 0.5 and assigned to a low-risk group when the probability was less than 0.5. Kaplan–Meier curves exhibited a distinct difference in the survival probability in the (A) Honghu subgroup and (B) Nanchang subgroup. The P-value was calculated using the log-rank test. [file Image_8.tif]
