# Supplementary material for: Development and Validation of a Deep Learning-Based Model Using Computed Tomography Imaging for Predicting Disease Severity of Coronavirus Disease 2019
Source: Front Bioeng Biotechnol. 2020 Jul 31;8:898. doi: 10.3389/fbioe.2020.00898 (PMC7411489; doi:10.3389/fbioe.2020.00898)
Supplement: Supplementary file 9 [file Table_1.DOC]

| Metrics | Architectures | | | | | | | |
| --- | --- | --- | --- | --- | --- | --- | --- | --- |
|  | ResNet34 | | DenseNet202 | | AlexNet | | VGG11BN | |
| Dataset( cohort)  Sensitivity  Specificity  Precision  False positive rate  False negative rate  Accuracy (%)  AUC  AUC 95% CI | Honghu  0.958  0.976  0.885  0.024  0.042  97.4  0.987  0.969-1.000 | Nanchang  0.875  0.785  0.714  0.215  0.125  81.9  0.892  0.828-0.955 | Honghu  0.917  0.957  0.800  0.043  0.083  95.1  0.956  0.911-1.000 | Nanchang  0.825  0.308  0.423  0.692  0.175  50.5  0.606  0.494-0.718 | Honghu  1.000  0.263  0.203  0.737  0.000  38.0  0.749  0.693-0.825 | Nanchang  1.000  0.000  0.381  1.000  0.000  38.1  0.569  0.456-0.682 | Honghu  0.875  0.784  0.433  0.216  0.125  79.9  0.920  0.871-0.969 | Nanchang  0.300  0.800  0.480  0.200  0.700  61.0  0.578  0.464-0.691 |

**Table S1. Performance metrics of disease severity prediction results under different network structures using CT imaging from the Honghu and Nanchang cohort.**
